# Between uncertainty and hope: Young leaders as agents of change in sustainable small-scale fisheries

**DOI:** 10.1007/s13280-021-01639-2

**Published:** 2021-11-02

**Authors:** Alejandro Espinoza-Tenorio, Romana Gabriela Ehuan-Noh, Gabriela Alejandra Cuevas-Gómez, Nemer E. Narchi, Dora Elia Ramos-Muñoz, Francisco J. Fernández-Rivera Melo, Antonio Saldívar-Moreno, José Alberto Zepeda-Domínguez, Juan Carlos Pérez-Jiménez, Alma Oliveto-Andrade, Jorge Torre

**Affiliations:** 1 Department of Sustainability Science, El Colegio de la Frontera Sur, Av. Rancho Polígono 2-A, 24500 Lerma, Campeche Mexico; 2 Comunidad y Biodiversidad A.C., Isla del Peruano No. 215, CP: 85448 Guaymas, Sonora Mexico; 3 Colegio de Michoacán, Cerro de Nahuatzen 85, Fracc. Jardines del Cerro Grande, La Piedad, Michoacán Mexico; 4 Department of Society and Cultura, El Colegio de la Frontera Sur, Carretera Panamericana y Periférico Sur s/n Barrio María Auxiliadora, 29290 San Cristóbal de Las Casas, Chiapas Mexico; 5 Universidad Autónoma de Baja California-Facultad de Ciencias Marinas, Carretera Transpeninsular Ensenada - Tijuana No. 3917. Colonia Playitas, CP 22860 Ensenada, Baja California Mexico

**Keywords:** Future, Influential youth, Leadership, Social learning, Social network, Sustainability process

## Abstract

The path to sustainable small-scale fisheries (SSF) is based on multiple learning processes that must transcend generational changes. To understand young leaders from communities with sustainable SSF management practices in Mexico, we used in-depth interviews to identify their shared motivations and perceptions for accepting their fishing heritage. These possible future decision-makers act as agents of change due to their organizational and technological abilities. However, young people are currently at a crossroads. Many inherited a passion for the sea and want to improve and diversify the fishing sector, yet young leaders do not want to accept a legacy of complicated socioenvironmental conditions that can limit their futures. These future leaders are especially concerned by the uncertainty caused by climate change. If fishing and generational change are not valued in planning processes, the continuity of fisheries, the success of conservation actions, and the lifestyles of young fishers will remain uncertain.

****Graphical abstract**:**

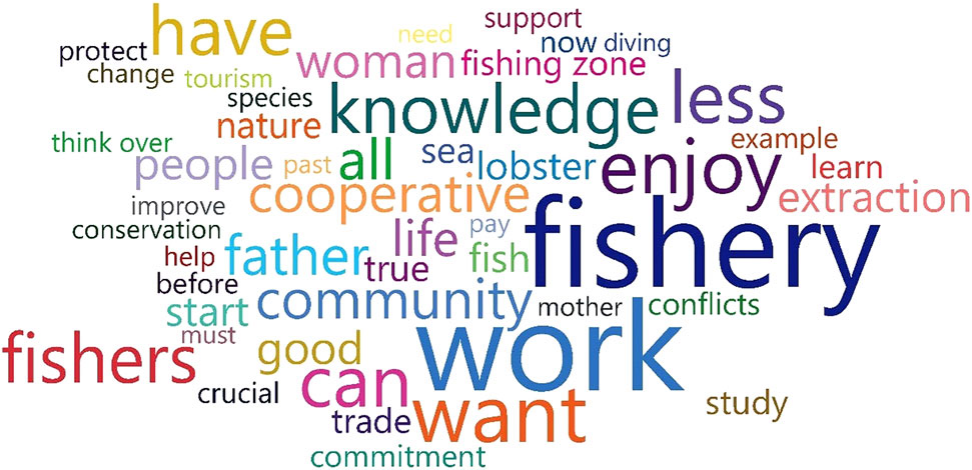

**Supplementary Information:**

The online version contains supplementary material available at 10.1007/s13280-021-01639-2.

## Introduction

The path toward the sustainable use of natural resources requires multiple, dynamic, and intergenerational learning processes (Zurba and Trimble 2014). Hopes for achieving sustainability lie in young people and their increasing degree of participation in decision-making processes (Yunita et al. 2018) and local governance (Oosterom 2018). Young people ultimately benefit from the natural resources currently being conserved (Clark et al. 2020) and have shown growing empathy and concern for both present-day and future challenges (Thew et al. 2020).

Youth is a sociocultural construct dependent on time and space (Durham 2000), although it generally refers to a stage in life in which concepts of identity, society, values, ideology, and citizenship are developed and adopted by an individual (Martínez 2008). Young people are capable of participating in transformative community and political actions and are seen as a productive social group (Moeller et al. 2018). The motivations of individuals (the drivers needed to conduct an activity) are partially the result of cognitive development and extrinsic agents (Gagné and Deci 2005; Pajares and Urdan 2006). Socio-emotional factors at the family level (Nokelainen et al. 2007; Perry 2009) and the influence of external actors (Navarro-Cendejas 2017) help shape the decisions of young people. These individuals find themselves at a crossroads between a worldview they inherit from their families and culture and the one provided by mainstream society through school-based education and the media (Ramírez-Pacheco et al. 2018). To understand how young people integrate themselves into the productive and civil sectors of society, it is crucial to understand how their actions result from an initial stage of wanting, which aligns with their aspirations, and a subsequent stage of power, which echoes their expectations and underlies their motivation to surround themselves with productive activity (Leavy and Smith 2010).

The activities of small-scale fisheries (SSF) are not limited to extraction; they encompass all activities undertaken by men and women at sea and on land that comprise the value chain (FAO 2018). Little is known regarding the many drivers that influence young people to uphold intergenerational continuity within SSF value chains (Power et al. 2014; White 2015). The knowledge, beliefs, traditions, and practices that develop as a result of the interactions between people and their environments (Berkes and Turner 2006) are what young fishers seek to complement with their own actions and perceptions (Harker-Schuch et al. 2021). This implies reevaluating their expectations and aspirations based on their unique trajectories. Thus, it is not clear how the links between new generations of fishers and the sea will be built or rearranged in the future (Coleman et al. 2019), particularly in the marginalized environments of developing countries (Tam et al. 2018; Arulingam et al. 2019).

**Fig. 1:**
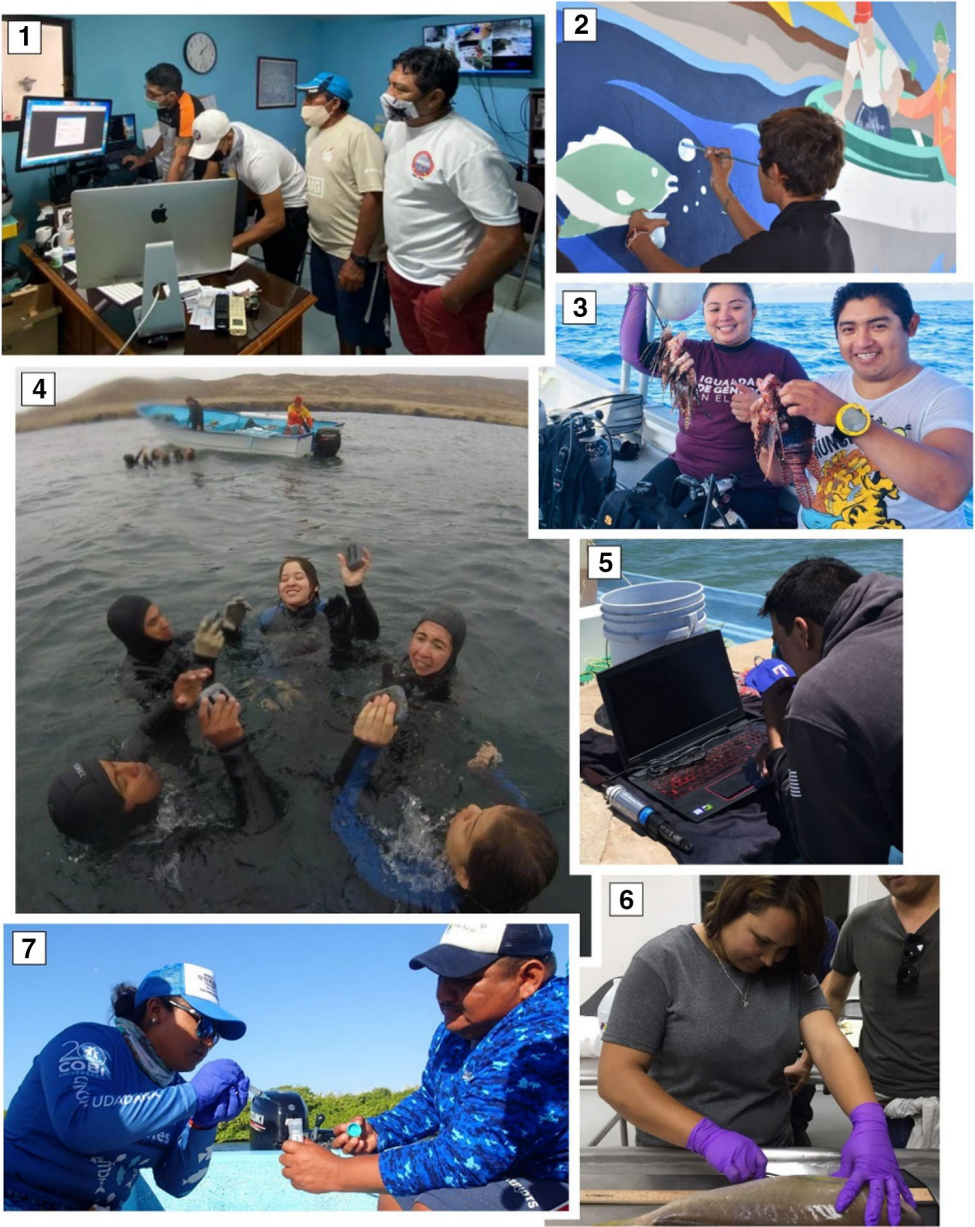
Young women and men participating in different activities associated with sustainable fisheries management in Mexico. **1** Training to program temperature recorders in the Mexican Caribbean. **2** Informative mural on the implementation of fishing refuge areas in the Gulf of California. **3** Young fishers involved in the Lionfish eradication of the Mexican Caribbean. **4** Dive training and certification in Baja California. **5** Oceanographic data collection in the Gulf of California. **6** Training in dissection procedures to obtain samples of *Caulolatilus princeps* in Baja California. **7** Water sampling for environmental DNA analysis in the Mexican Caribbean. **1** © L. Tamayo. **2**–**7** © COBI A.C

In the 1990s in Mexico, which is considered one of the top 20 fish producers worldwide, public fishing policies promoted sustainable fishing practices that resulted in timely advances in SSF management (Espinoza-Tenorio et al. 2011). Changes in national fisheries policies have had profound implications for the participation of young people in the entire fishery production chain. A new type of fisher, the young leader, has emerged with a new vision on how to live and take advantage of their fisheries management heritage (Fig. 1).

Leaders are often chosen for being superlative in traits, such as knowledge, trustworthiness, and prosociality (Von Rueden et al. 2014), and their potential to guide processes through influence over the community or acting as links with external relevant actors (Bodin and Crona, 2008); their relevance for fisheries governance have been clearly demonstrated, as they are recognized as one of the most important social-ecological factors for sustainability (Gutiérrez et al. 2011).

The objective of our research is to point out those factors that have carved out the character of young individuals determined to exert positive differential influence within their communities through leadership. Our study is based on in-depth interviews with young people (17–29 years old) who stand out for their abilities to lead, promote local self-organization, enforce rules, ameliorate attitudes associated with destructive practices, and resolve conflicts. These young leaders favor SSF sustainability in Mexico and may be future decision-makers. Their responses allowed us to examine the balance between aspirations and expectations and how these influence their motivations and perceptions regarding their roles in sustainable fisheries management. The results of this study will contribute to an international discussion on the future of SSF (Pauly 2018), but more importantly, this study aims to shine a light on the multigenerational consequences of global fisheries management policies that are often ignored by authorities (Neis and Williams 1996). If generational changeover is not investigated, addressed, and incorporated into planning processes, the pressure on aquatic resources is likely to increase (Biswas 2011), endangering both the continuity of fisheries (Neis et al. 2013) and the way of life of these young fishers.

### Sustainable fisheries management heritage in Mexico

Around 300,000 inhabitants in Mexico directly depend on SSF catches (CONAPESCA 2017). This number climbs to more than 2,000,000 when considering people who depend on the economic income and food provided by each link of the collective fisheries value chain (IMCO 2013). Most of these individuals live in rural coastal settlements (Espinoza-Tenorio et al. 2019) where a dependence on the marine environment is deep-rooted, and cultures have developed in which a fishing management heritage is passed on through the generations (Zepeda-Domínguez and Espinoza-Tenorio 2018).

An increasing demand for fish and their products in Mexico has led to overfishing, resource depletion, a loss of economic and human resources, and poor returns on investments (Fernández et al. 2010). Hence, civil society organizations (CSOs) and government agencies have collaborated with fishing communities to preserve marine resources and ecosystems while promoting the sustainable management of SSF. These alliances have allowed for improved compliance with regulations, a legitimization of the empirical knowledge held by fishers, and the promotion of ecosystem-based approaches to fisheries management (Fulton et al. 2019).

These collaborative efforts have shown varying degrees of consolidation. Communities in the Gulf of California and North Pacific stand out for the sustainable management of their highly productive fisheries (Espinosa-Romero et al. 2014). Communities in the Caribbean, which hosts the second-largest reef system in the world and constitutes a great development pole for tourism, have shown promising improvements in fisheries management (Ayer et al. 2018). In the Gulf of Mexico, fishing efforts have focused on the exploitation of finfish species, which operate simultaneously and unequally with efforts to extract hydrocarbons in shallow waters (Salazar-De la Cruz et al. 2020).

## Materials and Methods

**Fig. 2:**
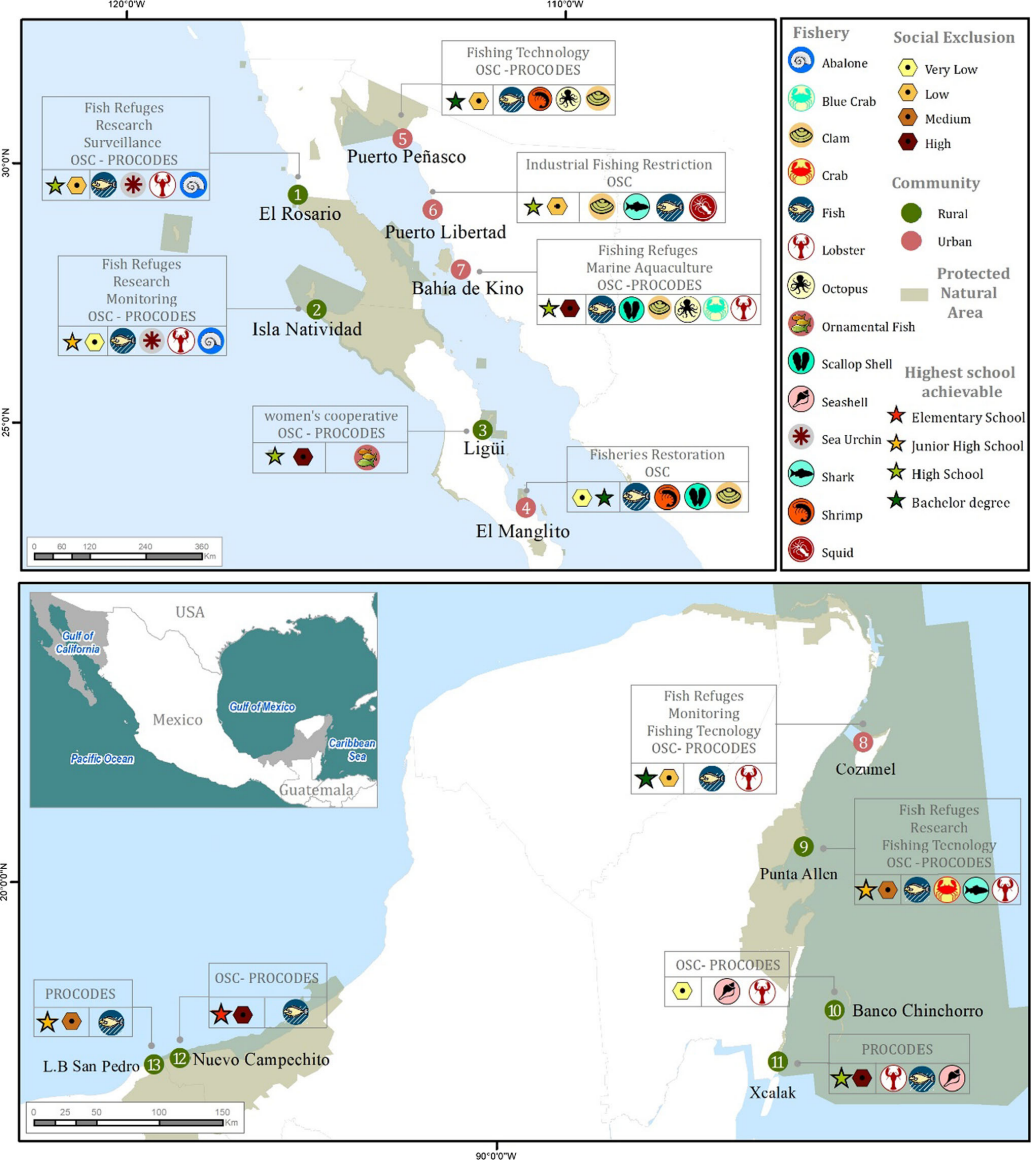
The locations and socioeconomic and educational descriptions of the localities in which the interviewees lived. The main fisheries and their sustainable management projects linked to Civil Society Organizations (CSOs) and the Program for the Protection and Restoration of Ecosystems and Species at Risk (PROREST) are presented. Figure created from the information obtained from the interviews, CONAPO (2015), and CONANP (2020)

A total of 15 men and 10 women from 13 locations in the Gulf of California, Caribbean Sea, Gulf of Mexico, and northern Mexican Pacific were interviewed for this study (Appendix S1). These young people are members of fishing communities, and their domestic units are economically associated with 14 SSF (Fig. 2), as either members of cooperative societies or as individual permit holders. Although some of these young people live in urban areas, they are mainly associated with rural populations with varying degrees of social marginalization, of which only five and three areas have the infrastructure for upper secondary education and higher education, respectively. All fishing communities in which the interviewees live have been supported by CSO projects and the National Commission of Natural Protected Areas (CONANP) and its Conservation Program for Sustainable Development (PROCODES) that have sought to implement sustainable practices and strengthen fisheries management.

To study future fisheries leaders, we used judgment sampling (Malterud et al. 2016) to purposively select informants in 2020 in different regional contexts given their specific age and gender. To include influential and highly motivated young people interested in involving themselves in management decisions (Gutiérrez et al. 2011), 12 adult leaders of cooperatives involved in sustainable fisheries suggested young people that met at least two of the following criteria: (1) they were members of families that supported and promoted sustainable fishing practices; (2) they were linked to the training efforts of CSOs and CONANP to develop and promote sustainable fisheries; and (3) they worked in a link of a productive fisheries value chain subject to sustainable management practices (e.g., in fishing refuges or as survey divers conducting citizen science). Both interviewees and adult leaders were provided with all the pertinent information pertaining to this study (i.e., the objectives, goals, funding information, possible risks and benefits, and contact information).

In September and October 2020, virtual interviews were conducted through video conferences and telephone calls due to the Covid-19 pandemic. The ethical standards of informed participation of the Code of Ethics of the International Society of Ethnobiology were followed (ISE 2006). Participation in the study was voluntary. Interviewees were given the option to reserve their answers to specific questions, refuse to participate, or stop participating at any time without providing a reason and without any associated sanctions or consequences. Approval was obtained to record the interviews, and consent was obtained from the guardians of any minors.

### In-depth interview

A mixed questionnaire was divided into seven sections (Appendix S2). Close-ended questions in sections I–III were used to characterize the interviewees and their family environments. Sections IV–VI were semi-structured to capture agreements and disagreements concerning aspirations and expectations as well as real-life fishing stories to identify the motivations for embracing their fishing management heritage. Aspirations (i.e., utopian and idealized longings) are understood to be what one wants to become or have and are linked to an ideal of what life should be, while expectations (i.e., realistic evaluations of probabilities) differ in that they are founded on lived reality. To evaluate aspirations and expectations, the reference framework used by Patton and Creed (2007) was adopted. This proposes a method for observing perceived barriers and identifying potential discrepancies between two possible life projects and their subsequent consequences.

In section VII, the perceptions of young people regarding the processes that favor SFF sustainability were identified. An infographic was designed (Appendix S3), and the scale of maximum difference (MaxDiff; Louviere et al. 2015) was used to determine the level of importance attributed to each of the eight processes stemming from voluntary guidelines to achieve sustainability in SSF (FAO 2018). Each young person was asked to explain the progress and challenges associated with the three processes that they considered to be the most relevant in their fishing communities.

### Analysis

Answers to each question were transcribed and categorized in Atlas.ti 8 (ATLAS. ti Scientific Software Development GmbH, Berlin, Germany) to identify information patterns (e.g., topic frequencies; Saldaña 2013). Categorized responses of the interviewees were analyzed with SankeyMATIC (Bogart 2018), and flow diagrams of internet use and the stories of young people were constructed according to three age stages: childhood (< 10 years), adolescence (10–21 years), and young adult (22–29 years). Atlas.ti was also used to identify the 50 most frequently used words by the interviewees, which were used to build a word cloud (see Graphical Abstract).

Standard deviation (SD) and range are shown as a measure of variability. A t-test was used to test whether there were differences in age between young men and women. Independent test was conducted to test for independence between genre and educational level.

## Results

People interviewed in this study were 24 years old on average (SD = 3.4; range 17–29 years). The age of men ($\overline{\chi }$= 24, SD = 3.3) and women ($\overline{\chi }$= 24.1, SD = 3.7) was not statistically different (*t* = − 0.07, *P* = 0.94). Of these, 14 people were single and lived with their parents, five people were married, and six people were in common-law partnerships. Only three of the single men lived on their own, and nine people were parents. The women ($\overline{\chi }$ = 19.5 years, *n* = 2) became mothers before the men became fathers ($\overline{\chi }$ = 21.3 years, *n* = 7).

Thirteen interviewees said that they obtained medical care from the Mexican Social Security Institute, either because they had access to this system as students or because their parents were affiliated in some way. When young people conclude their studies, stop studying, or emancipate from their parents, they often use low-cost private clinics offered by pharmacy chains to cover their health needs and occasionally turn to traditional medicine or the free public system.

### Generational change

**Fig. 3:**
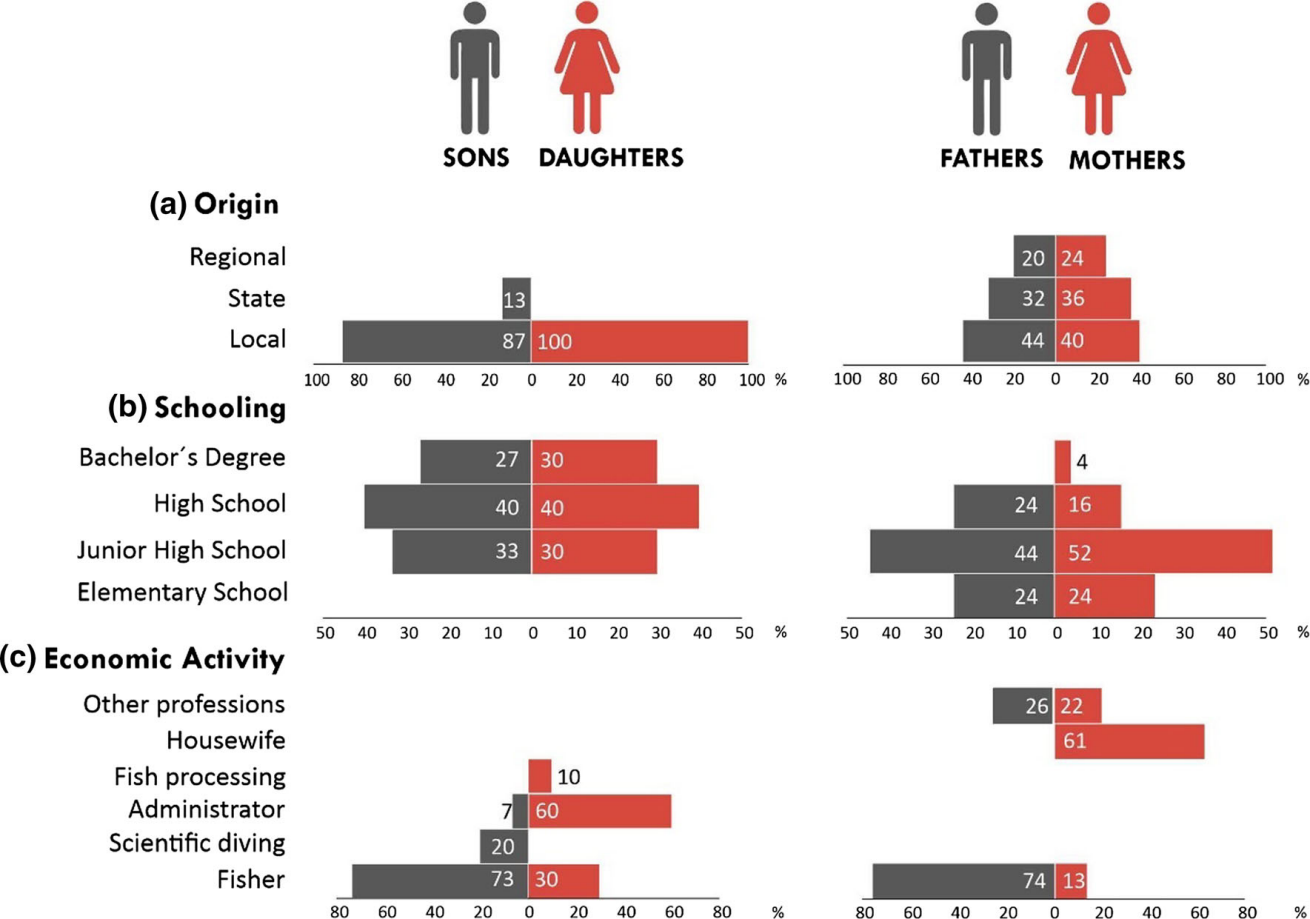
Comparison of the profiles of the interviewees (i.e., sons and daughters) with those of their fathers and mothers who participated in sustainable fishing in Mexico. Gender comparison among men (gray) and women (red), including their geographical origin, educational level, and main economic activity. Figure created from the information obtained from interviews

Most of the interviewees were originally from the locality in which they now work (Fig. 3a). The fathers and mothers of the interviewees, on the other hand, had migrated from other localities in the same state or from other states within the region.

The interviewees went to high school, and some obtained technical specializations (e.g., ecotourism; Fig. 3b). Almost a third of the interviewees began undergraduate studies, and some completed them. In contrast, their fathers and mothers mostly attended high school, except for one mother in the Caribbean. Educational level is statistically independent of gender in young people (*X*^2^ = 0.04, *P* = 0.98) and their fathers and mothers (*X*^2^ = 1.5, *P* = 0.67). According to the people interviewed, they studied because their parents worried about the problems inherent to fishing, such as economic instability or being away from home for long periods.

Despite the evident family support toward studying, half of the interviewees interrupted their education, either voluntarily or by dropping out of school (four men) due to a lack of money, uncertainty regarding what to continue studying, or little interest. A young Caribbean fisher (C-BC2) who became a father at 19 years of age dropped out of high school due to “the poor financial stability of my parents, and […] we made the mistake of starting to earn money; we worked outside of school and then the ambition for money won me over.” The women who abandoned their studies left due to limited financial resources and sometimes due to the lack of family support.

Interviewees participated in more fishing sectors than their parents, who also worked in other professions (Fig. 3c). However, a labor bias driven by gender and age was present. Younger and older men mainly worked extracting fishery resources, while young women mainly worked in the administration of fishing organizations. The mothers of the interviewees primarily worked as housewives, in other economic activities, or in fishing but without direct economic remuneration (C-X1). Therefore, economic activity depends on gender in young people (X^2^ = 10.3, P < 0.01) and their fathers and mothers (*X*^2^ = 23.8, *P* < 0.0001).

#### The new digital tools

**Fig. 4:**
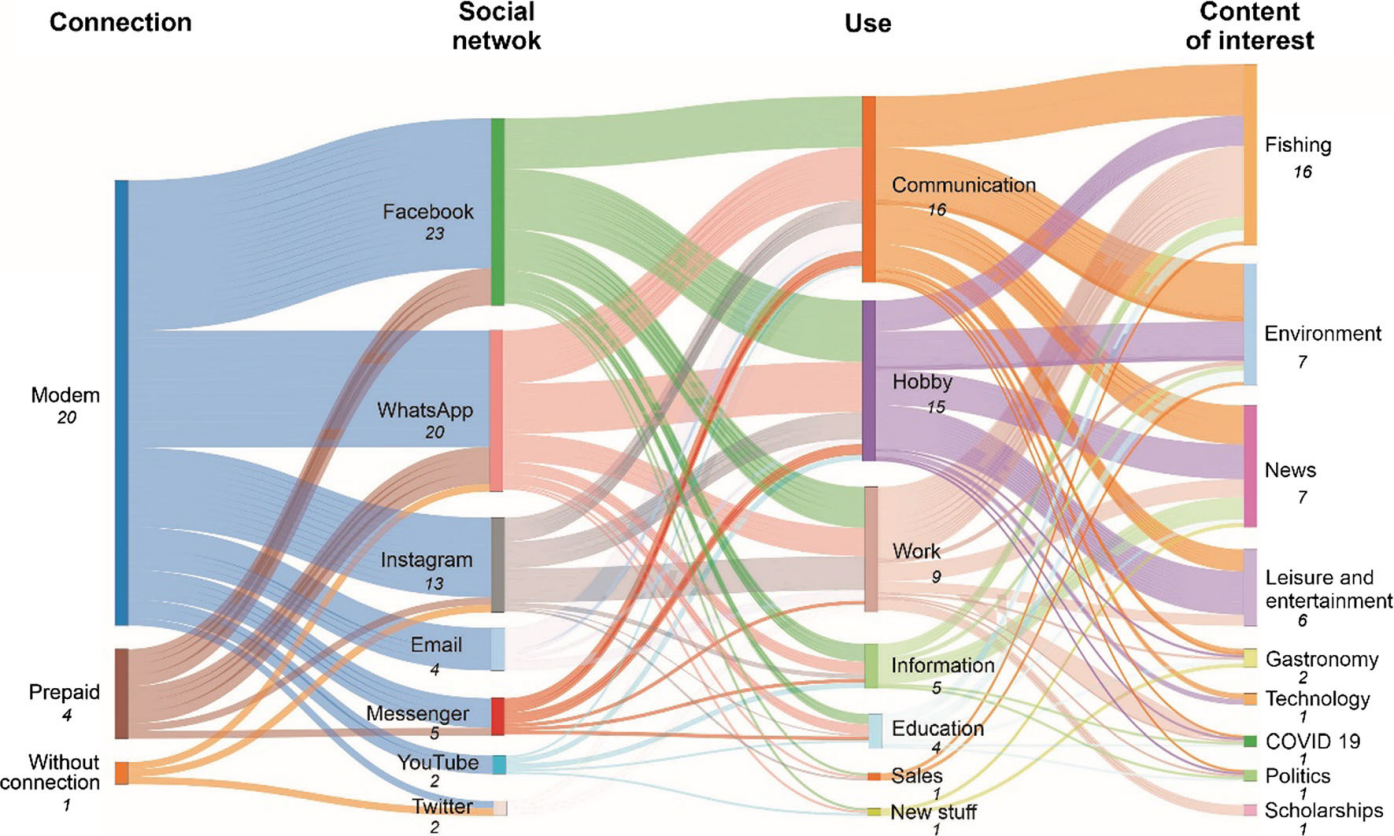
Internet access and the use of social networks among the young people interviewed who participated in sustainable fishing in Mexico. The number in italics indicates the number of answers that were the same among interviewees

Twenty interviewees said they accessed the internet using modems from communication companies or from public or community satellite dishes in rural areas (Fig. 4). Four interviewees used a prepay system and only one young woman (GC-L) from an isolated location did not have a permanent internet connection and often traveled to access one.

On the Internet, people use social networks, such as Facebook, WhatsApp, and Instagram, as hobbies and a means of communication with family and friends. Additionally, these social networks are used to promote and coordinate the activities of the organizations in which they work. These platforms and email provide access to nine different categories of content, including fishing information, environmental news, news in general, and leisure and entertainment topics.

### The intricate path to becoming a fisher

**Fig. 5:**
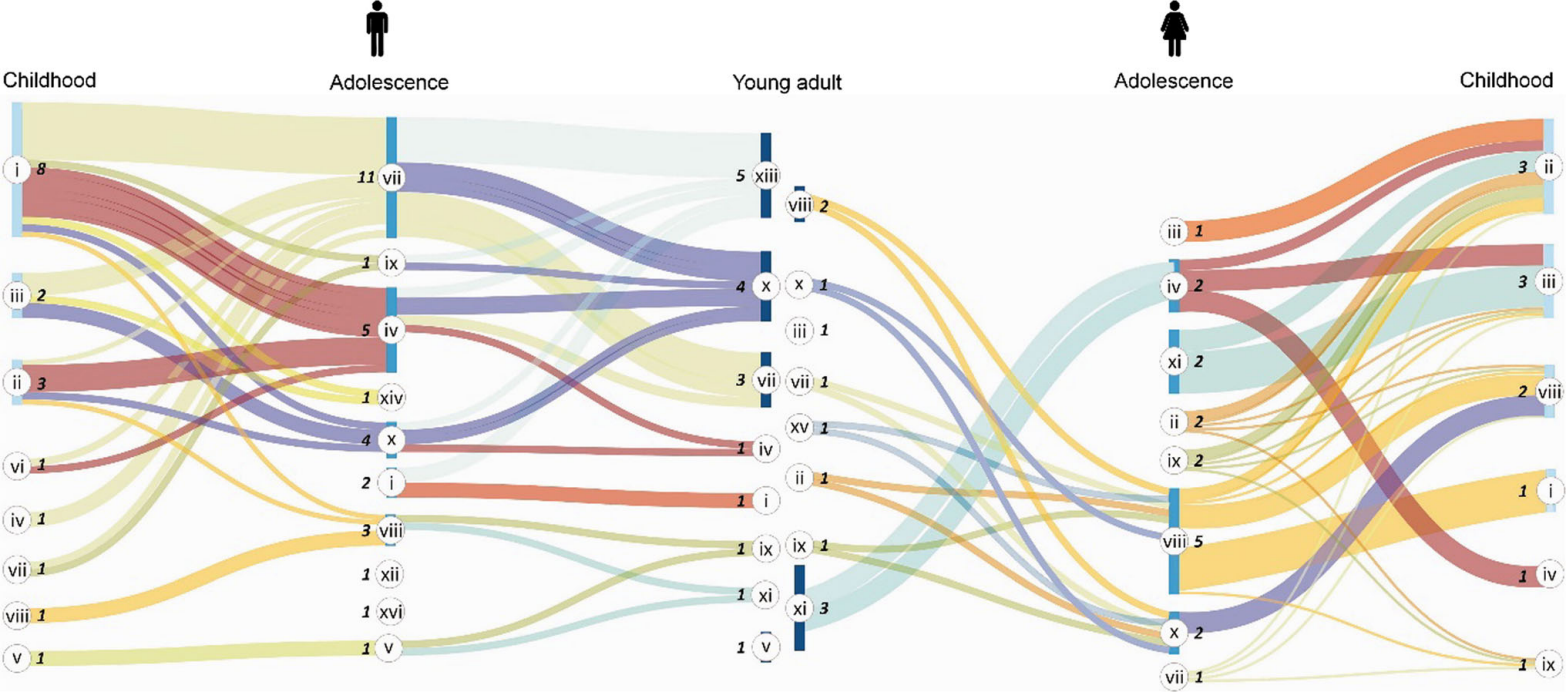
The trajectories of young fishers in Mexico and their staggered participation in the fisheries: (i) apprentice companion, (ii) coastal fishing, (iii) recreational fishing, (iv) eventual companion, (v) embarking/disembarking, (vi) beach cleaning, vii) waiter/fishing companion, (viii) processing and (ix) selling fishery products, (x) survey diver, (xi) administrator, (xii) trainer, (xiii) captain, (xiv) sport fishing, (xv) cooperative leader, and (xvi) aquaculturist. The Arabic number in italics indicates the number of answers that were the same between interviewees. Figure created from the information obtained from the interviews

From a review of the fishing activities that the interviewees conducted since childhood, it was determined that they participated in 16 activities within 12 fisheries (Fig. 5). Men and women followed different staggered and gradual trajectories to learn the trade. During childhood, the interviewees reported participating as apprentice companions or in recreational fishing for household consumption in coastal protected places like lagoons and beaches. At the beginning of adolescence, the type of fishery work generally changed depending on whether someone was a man or woman. While young women mostly worked processing fishery products, young men became apprentices (waiter/fishing or eventual companion), which is a decisive step toward extracting fishery resources. In this period, young people also moved away from fishing activities to attend school, which sometimes implied leaving their rural communities.

As young adults, most men became boat captains or survey divers, while coeval women of the same age were primarily positioned in administrative posts or in processing-related activities. Many women take on these jobs after having studied, regardless of fishing experience. Additionally, some women carry out these activities because they married a fisher.

#### Aspirations vs expectations: a crossroads between the dream and reality

A successful life in fishing for young people is not guaranteed, as real opportunities and their ideals interfere with their destinies as young fishers. The main benefit that the interviewees perceived from working in fishing was the possibility of obtaining food, although men tended to also value economic income, while women valued the knowledge they obtained of natural resources and the opportunities for paid work. Both men and women appreciated the benefits obtained by belonging to a cooperative (e.g., medical insurance) and the way of life in their communities.

The interviewees were also interested in fishing because they enjoyed life near the sea and coast, including the landscapes, food, opportunities for relaxation, and knowledge that this lifestyle provides. In addition, the interviewees also valued the potential for adventures, social connections, and personal challenges that come from being a working fisher. A free diver from the coral reefs of Banco Chinchorro (C-BC2) said that his activity “…is an unbeatable [and] indescribable experience because the sea gives us a new experience every day.” The interviewees did not like fishing that was economically unstable or, as a young man who works in ecotourism considered (C-A4), fishing without restrictions, which can result in “decreases in the populations of animals in the ecosystem.”

Young people who view fishing as an ideal activity have histories of participating in fishing since childhood and have not sought other job opportunities, as they consider that there are ample opportunities within the value chain like those within the lobster fishery in the Caribbean Sea, which is perceived as having viable fish stocks and high levels of community organization. If it becomes challenging to continue fishing in their places of origin, the interviewees planned to achieve their aspirations through activities complementary to fishing that offered improved economic autonomy and personal satisfaction (i.e., professionals or environmental activists). A young engineer (C-X1) in environmental sciences who was single and the daughter of a Caribbean fisher said that although she would like to live in cities with greater services and safety, fishing is “… not something that could be left behind.”

Some young people continue fishing for personal satisfaction, although they have little hope of continuing in the activity. This crossroads is due to the high degree of uncertainty in the fishing sector, such as the collapse of the pen shell (*Atrina tuberculosa*) and catarina scallop (*Argopecten ventricosus*) fisheries or greater fishing restrictions in the Gulf of California. In the absence of fishing, the interviewees would consider engaging in non-fishing activities and taking advantage of regional opportunities, such as those related to tourism services (e.g., receptionists and cooks).

Other interviewees would also leave fishing if they found a job that allowed them to obtain sufficient and stable salaries. These young people only participate in fishing due to a lack of better jobs or because they have been affected by crises in other productive sectors (e.g., the hydrocarbon industry). It is common for these young people to have pursued the professional careers available in their region (e.g., those related to administration, tourism, and engineering), although possibly not the ones they would have liked, such as architecture or international relations. Occasionally, acquired skills support local SSF (e.g., administration and being technologically savvy), even without fishing jobs being the first choice of young people.

### Living with what is necessary and restoration

The interviewees relate sustainability to conservation and the responsible use of fishing resources. For a young descendant of farmer-fishers (C-BC1), the message is clear, “*live using only* what is necessary.” In addition, given the evident deterioration of the fisheries, the interviewees saw the recovery of resources as an integral part of sustainability, saying "we are going to make use of a resource, but we are going to do something to make it return to a level that is the same or even better" (GC-M1). The interviewees showed greater concern for fisheries due to risks associated with disasters and climate change, although other concerns were also present (Table 1). However, priorities differed between genders, and the ten women interviewed had more concerns related to sustainable resource management, governance, and gender equality.

**Table 1:** Weighted importance assigned by young fishers to sustainable fishing processes

Priority	Process	Score		
		Man	Women	Total
1	Disaster risks and climate change	**5.9**	3.8	**5.0**
2	Sustainable resource management	4.5	**5.3**	4.8
3	Information, research, and communication	5.3	3.9	4.7
4	Value chains, post-harvest, and trade	4.6	4.7	4.6
5	Policy coherence, institutional coordination, and collaboration	4.5	4.3	4.4
6	Responsible governance of tenure	3.7	5.2	4.3
7	Gender equality	3.7	4.8	4.2
8	Social development, employment, and decent work	3.9	4.0	3.2

The highest scores for each category are in bold

#### The worst is yet to come

The visions of the future for the interviewees included climate change as the greatest external threat to fishing since it may threaten the ability to continue fishing. The interviewees were aware that “climate change […] affects or benefits, but more than anything it affects” (P-R3), and they had concerns about coral bleaching, changes in marine currents, and the increased risk of hurricanes. It is “… as if the planet was withering away” (C-BC2).

The interviewees recognized local strategies to mitigate the possible impacts of climate change in marine communities. However, they perceived that improved awareness and community training are needed since this issue does not receive the degree of attention it requires while concrete measures to mitigate disasters are lacking.

#### Dialogue and consensus

The interviewees were aware of the efforts made by their cooperatives to implement responsible fishing practices, but they were also aware that most fishers sometimes cannot respect closed areas or periods because “they live day to day and need money to live” (C–C). In addition, although fishers are kept informed through general assemblies, most prefer to simply deliver their product and fully delegate the responsibility for its sale, administration, and dealings with the authorities to cooperative leaders. As such, given the excessive bureaucracy and intersectoral lack of coordination, it is common for people to distrust the authorities.

The interviewees suggested that the problems that persist in their communities may be addressed through dialogue with government authorities. Management based on organization was summarized by one person who said "if everyone were organized, there would be no fishers to obtain the product" (GC-BK1). This dialogue would allow the fishing organizations to overcome scenarios of apathy, fines, punishments, or conflicts.

#### You must learn before you extract

According to the interviewees, it is necessary for all generations of fishers to receive training on the rational use of resources and climate change, advice on how to value the way of life of fishing families, and information on local, sustainable management practices for resource utilization and conservation. For several of the respondents, this basic knowledge implies understanding how to work as a team, sell the product (e.g., knowing the price of the product in international markets), operate within the laws that regulate fishing, collaborate with institutions, and work with a focus on gender equality in all areas. To achieve this, fishers must commit to learning and understanding sustainable fishing. In addition, the interviewees identified an essential element of sustainability in social co-responsibility and the need to disseminate information among consumers and themselves.

#### Less catch, more value

For the interviewees, the future of the market is “where less product is produced, but it is worth more” (P-R2). There are fisheries in the Caribbean, the Gulf of California, and the North Pacific that have moved toward sustainability, expanding their value chains to a predominantly international market. In these fisheries, young people are satisfied with their profits, as a young lobster fisher (C-BC2) mentions, "…with exports they pay us much better than with local trade." In addition, young people do not have a good view of the national markets because they distrust intermediaries, as “they do not offer a legal guarantee of payment” (GC-PL2).

Interviewees wish for SSF to benefit from the market by diversifying and creating new processes that add value to their products, eliminate intermediaries, and improve resource management. To do this, the interviewees proposed avoiding ineffectual administrations, having human resources with the necessary knowledge to export the products, and acquiring storage equipment to process and pack products. The interviewees also proposed strengthening electronic marketing strategies with web pages and social networks.

#### Inspection and support

For the interviewees, inter-institutional coordination and collaboration are essential for promoting a consensual work program among fishers, government authorities, and CSOs. One limitation is the centralization of governmental institutions and elaborate procedures, which results in a lack of dialogue among the interested parties, as there are no local officials that have been empowered to develop or establish agreements. In some regions, the implementation of conservation programs oriented toward surveillance has resulted in territorial conflicts. For example, interviewees from the Gulf of California mentioned critical family changes when they had to move away from their fishing areas due to the establishment of no-fish zones (i.e., protected areas, fishing refuges, and community reserves). As they witnessed the socioeconomic implications that these changes caused, interviewees were concerned that if illegal fishing and the over-exploitation of fishing resources continued, closed periods and no-fishing zones would be increased and expanded. "There is going to come a time when there is an eternal ban; you will not even be able to fish," emphasizes a diver and fisher from the Gulf of California (GC-BK3).

#### The sea for those who care for it

According to an adolescent daughter of a fisher (GC-PL1), law enforcement should be equitable, and it is "… the responsibility of the fishers to respect them [the laws] and the responsibility of the government to sanction [offenders]." However, the right to access and use their fishing areas is still a matter of concern for young people. The interviewees pointed out that even though taxes are paid to the government to be able to sell fishery products, their rights are violated daily as there is no security in their communities and illegal fishing is not properly addressed (e.g., respecting the anonymity of complaints) while mechanisms have not been put in place to guarantee fair and equitable fees for their products.

The young interviewees proposed that a commitment to responsible fishing should be an unconditional requirement for everyone who wants to enter the fishing sector. By knowing and understanding their rights and responsibilities, it is possible to prevent the dispossession of territories that form part of their fishing heritage. In the words of the son of a fisher (C-A4) “…the sea is for those who really know how to take care of it […]; if I don't know how to take care of it, sustainably use it; I should not have that right, although someone who does value it should.”

#### Same job, same pay

According to the interviewees, although the prioritization of gender equality is different between young men and women, they receive the same pay for the same tasks, regardless of gender. Men consider that women who participate in extraction activities, although they are still very few, deserve admiration and support. Moreover, all women were proud to take on an activity that has been historically linked to men. "Being a woman makes me proud because the truth is I'm good at fishing," says a fisher from the North Pacific (P-R1). Being a woman survey diver or engaging in the direct extraction of fishery resources represents an achievement considering the limits imposed on their mothers and grandmothers. However, some of the interviewees felt that they are still not considered in the decision-making process. For them, men still predominantly occupy jobs within cooperatives. Even in administrative positions, women are yet restricted to performing minor tasks and are paid less.

#### How to live from fishing

The demands of the interviewees were linked to an overall lack of conformity regarding the needs of their communities. Despite constituting an important productive sector of the nation, fishing communities have not yet been provided with basic rights and services (e.g., public safety, health, housing, and education). In some communities, water, electricity, and access to the internet have had to be provided by the community members themselves. The growing insecurity for fishers at sea, drug addiction, and the threat of organized crime have not been addressed. Fishing must be valued and promoted so that young people see it as a relatively quick way to earn money. In some places, such as the Caribbean, young people have complemented fishing activities with ecotourism to compensate for declines in their income during closed seasons or during periods when catches are low.

## Discussion

The sustainability of SSF has been built on lessons learned, successes, and failures (Salas et al. 2019). Achievements in sustainable fisheries will endure over the long run if they are maintained, enriched, and expanded upon by future generations (Berkes 2003). Therefore, maintaining the progress that has been made will largely depend on continuing efforts from young people to propose and implement actions to alleviate or reverse environmental degradation. New generations of fishers will inherit a complicated legacy that they must decide to adapt, assimilate, or deny given a highly uncertain future. Nowadays, young fishers operate in new and complex contexts with different knowledge, tools, and motivations than those of their parents.

The interviewees look forward to maintaining coastal livelihoods, and their hopes lie in continuing to participate in SSF. They are heirs of a fishing culture rooted in teaching children about the sea (Malm 2015). Thus, like their predecessors, young fishers constantly adapt to the changing conditions that are inherent to the seas and oceans (Tolentino-Arévalo et al. 2019) and to the complexity and heterogeneity of SSF (Coronado et al. 2020). Due to the relationship between biocultural heritage and the identity of individuals (McRuer and Zethelius 2017), the territories in which young people grow and their histories influence their opportunities to achieve long-term goals. For this reason, young people are strongly influenced by socioenvironmental contexts, and their vision of sustainability reflects the different needs of each region and fishing community.

Fisheries management heritage is not fixed. It will always be necessary to integrate new knowledge, systems, technologies, and ways of dealing with changing socioenvironmental conditions. Therefore, young people face socioenvironmental challenges when creating opportunities, diversifying their activities, or adopting new practices (Glover and Sumberg 2020). The new generation aspires to both fish and carry out other activities, achieving a balance between their aspirations and expectations in the places they want to live. Young people add new empirical and scientific knowledge and tools to the skillsets of their parents. These additions allow them to identify problems that previous generations were slow to address. For this reason, young people are essential for innovation and diversification (Zurba and Trimble 2014). During the COVID-19 pandemic, fishers have shown a great capacity to technologically adapt to lockdown measures and market closures (Lopez-Ercilla et al. 2021). Young people played a key role in helping their parents learn to use digital media and videoconferencing software, the internet, and social media to participate in electronic commerce.

In emergent economies like Mexico, SSF face complex challenges when adapting to the new conditions of international markets, the increasingly strict regulations for fishing activities, a lack of basic rights and services, and general socioenvironmental vulnerability. This has generated an ambivalence in young people between frustration and hope. The frustration of not fulfilling their aspirations was notable among the 25 young people included in this study. The interviews with young women and men revealed that educational levels have improved compared to those of their parents. In addition, the gender gap in fisheries has reduced, cooperative work is appreciated, and digital capabilities have opened new doors. However, young people continue to face precarious conditions, including interrupted education, a lack of preferred work in fisheries, a lack of health care and services, and the relegation of women to performing minor tasks.

Competing under disadvantageous conditions in the labor market creates conditions of hopelessness, which may even be greater among young people who are part of SSF and who are not yet part of sustainable practices. In the rest of the country, most young fishers share a deep sense of belonging to a fishery, but they are growing up under more adverse socioeconomic conditions than those who were interviewed in this study (e.g., no social security; Fernández-Reyes 2018), and thus, participation in SSF is unappealing to them (Castro-Mondragon et al. 2015). Nonetheless, fishing constitutes the only labor source that many young people in rural zones have (Coleman et al. 2019). One of the young interviewees clearly described this unattended issue: "…unfortunately most of the people who enter […] fishing do so because they no longer have any other option" (C-BC1).

In addition, the gerontocratic systems still present within fishing organizations limited participation of young people in decision-making processes, hindering the integration of innovative fishing practices (Zepeda-Domínguez and Espinoza-Tenorio 2018) or bidirectional learning. Under these circumstances, activities designed to foster exchanges among fishers are particularly important because they provide opportunities for fishers to share the lessons, knowledge, and skills they have acquired while exploring new ideas that will allow them to learn, adapt, and strengthen their understanding (Jenkins et al. 2017). Although there is a lack of interest in formal education, learning from experience and the information that is connected through different contexts is valued by young fishers. As in other societies that survive by collecting and hunting (Lew-Levy et al. 2017), it is through these empirical learning spaces and by learning about other regions that fishers can identify possibilities for transformation. In doing so, they gain the opportunity to anticipate situations and design preventive or alternative measures before negative impacts occur in their communities.

### Youth priorities for sustainability

Although all axes for sustainable fishing are equally important, the interviewees, like other young people in the world (Harker-Schuch et al. 2021), were especially concerned with the environmental future of their coastal zones. These areas are vulnerable to extreme natural phenomena, especially in emerging countries with precarious coastal infrastructure (Reguero et al. 2015). With rapid demographic changes and low incomes, the economies in these areas are mostly dependent on natural resources (Maja and Ayano 2021).

For the interviewees, the key to improving fisheries management lies in knowledge and organization. The interviewees shared a collective identity that was reflected in their aspirations to become part of a cooperative, which is related to the axes of governance and dignified employment. Young fishers want to break down communication barriers and bridge the communication gap between authorities and fishers.

The hierarchical organization of priorities differs between genders. The ten women interviewed assigned greater importance to the issue of gender equality than did the men. Women had an optimistic view of the future, as their experiences seem different from those of their mothers. Young women influence administrative aspects and do so with a higher level of education and to a greater extent than do men. However, creating mechanisms that women can use to become active participants in decision-making processes must be prioritized (Solano et al. 2021).

## Conclusion

Young fishers are the engine of transformation in fisheries management, although they are still invisible to the fishing system in practical terms. In Mexico, young people represent 24.6% of the total population (INEGI 2020) and ignoring them is to discard a real agent of change. Society will need to quickly adapt to their vision of the future and recognize the problems they face. Acknowledging the contributions and efforts of young people in SSF will generate opportunities to improve sustainability and living conditions. Therefore, to bridge the gap between the aspirations and expectations of young people for becoming effective agents of change and sustainability, they must be included in decision-making processes and spaces must be created for them to exert their power. Society will also have to truly value the fishing trade, by establishing fishing as a decent and highly valued job within communities for young people so that their needs and those of their families may be met (Larson et al. 2018). However, young men and women are not the same; as we have shown, they have different interests and require different support.

In the transition from one generation to the next, fisheries institutions must adequately prepare and support men and women to claim a way of life that is fundamental for conserving marine environments and fostering food security. Building spaces for hope (Harvey 2000) has become a central component of organization, social participation, and environmental awareness strategies. However, failure to do so may put SSF at greater risk, both in Mexico and elsewhere.

As such, it is important to consider young people as future community and fisheries leaders. This poses significant challenges for the design of public policies capable of effective responses, which must comprehensively address challenges related to decision-making, fair and equitable labor dynamics, and overall opportunities that currently limit the potential well-being of young fishers. Therefore, it is not only the management and inclusion model that must change but also the accumulation model as well. A new accumulation model in which fishing cooperatives can reinvest part of their profits in shares and institutions that strengthen the social capital of their communities is needed (Barkin and Rosas 2006). It is essential to understand young people in the context of educational, labor, and research policies to see them as agents of social transformation and open for dialogue. Governments, CSOs, and researchers must redouble their efforts to incorporate, train, and understand young men and women as future decision-makers in more comprehensive and profound ways than before.

## Supplementary Information

Below is the link to the electronic supplementary material.

Supplementary file1 (PDF 1928 kb)
