# Supplementary material for: Between uncertainty and hope: Young leaders as agents of change in sustainable small-scale fisheries
Source: Ambio. 2021 Nov 2;51(5):1287–301. doi: 10.1007/s13280-021-01639-2 (PMC8561361; doi:10.1007/s13280-021-01639-2)

Ambio

Electronic Supplementary Material

This supplementary material has not been peer reviewed.

Title: **Between uncertainty and hope: young leaders as agents of change in sustainable small- scale fisheries**

Authors: Alejandro Espinoza-Tenorio, Romana G Ehuan-Noh, Gabriela A Cuevas-Gómez, Nemer E Narchi, Dora E Ramos-Muñoz, Francisco J Fernández-Rivera Melo, Antonio Saldívar-Moreno, José Alberto Zepeda-Domínguez, Juan Carlos Pérez-Jiménez, Alma Oliveto- Andrade, Jorge Torre.

Appendix S1

Youth interviewed whose life stories have developed in parallel with community process in favor of PPE in Mexico.

Region	Location	ID	Age	Civil Status	Sex/Gender	Birthplace	Children	Paternity (age))	Education	Ethnicity	Health services
North Pacific	1. El Rosario de Arriba	P-R1	23	Married	Woman	Local	0	-	High School	No	Pharmacy
		P-R2	23	Married	Man	Local	2	19	Unfinished Bachelor´s Degree	No	Pharmacy
		P-R3	28	Free union	Man	Local	1	27	Junior High School	No	IMSS/ISSSTE
	2. Natividad Island	P-IN	24	Married	Man	Local	1	22	Unfinished Junior School	No	Pharmacy
Gulf of California	3. Puerto Peñasco	GC-PP	21	Single	Woman	Local	0	-	High School	No	IMSS/ISSSTE
	4. Libertad Port	GC-PL1	17	Single	Woman	Local	0	-	Junior High School	No	Pharmacy
		GC-PL2	29	Free union	Woman	Local	0	-	Bachelor's Degree	No	Pharmacy
	5. Kino Bay	GC-BK1	20	Single	Man	Local	0	-	High School	No	IMSS/ISSSTE
		GC-BK2	24	Free union	Man	Local	1	23	Unfinished Bachelor´s Degree	No	INSABI
		GC-BK3	27	Free union	Man	Local	4	21	Junior High School	No	IMSS/ISSSTE
	6. Ligüi	GC-L	27	Married	Woman	Local	0	-	Bachelor's Degree	No	INSABI
	7. El Manglito	GC-M1	23	Single	Woman	Local	0	-	High School	No	IMSS/ISSSTE
		GC-M2	23	Free union	Woman	Local	1	20	Junior High School	No	INSABI
Caribe	8. Cozumel	C-C	29	Single	Woman	Local	2	19	Bachelor's Degree	No	IMSS/ISSSTE
	9. Punta Allen	C-A1	19	Single	Man	Local	0	-	High School	No	Pharmacy
		C-A2	19	Single	Man	Local	0	-	Junior High School	No	IMSS/ISSSTE
		C-A3	23	Free union	Man	Local	1	18	Junior High School	No	IMSS/ISSSTE
		C-A4	26	Single	Man	Local	0	-	High School	No	Traditional medicine
		C-A5	26	Single	Woman	Local	0	-	Junior High School	No	IMSS/ISSSTE

	10. Banco Chinchorro	C-BC1	27	Single	Man	Local	0	-	High School	No	IMSS/ISSSTE
		C-BC2	29	Married	Man	Local	2	19	Unfinished High School	No	IMSS/ISSSTE
	11. Xcalak	C-X1	23	Single	Woman	Local	0	-	Bachelor's Degree	No	Farmacia
		C-X2	24	Single	Man	State	0	-	Bachelor's Degree	No	IMSS/ISSSTE
Gulf of Mexico	12. Nuevo Campechito	GM-NC	27	Single	Hombre	Estado	0	-	Bachelor's Degree	No	Privado
	13. San Pedro	GM-SP	20	Single	Hombre	Local	0	-	High School	No	IMSS/ISSSTE

IMSABI=Instituto de Salud para el Bienestar; IMSS=Instituto Mexicano del Seguro Social; ISSSTE=Instituto de Seguridad y Servicios Sociales de los Trabajadores del Estado

Appendix S2
Survey form

CHARACTERIZATION OF THE PROFILE OF YOUNG PEOPLE IN FISHING ACTIVITY

Interview

Good morning my name is ________________________________ and I collaborate with the organization Community & Biodiversity, A.C. (COBI) and School of the South Frontier (ECOSUR). The interview is part of the research project "Young people in fishing: the new generation of fishermen" that aims to identify and characterize young people from different communities who are involved in the activities of the fishing sector. Your participation is voluntary and the information you provide will be treated confidentially; Your name, opinion and other information provided will only be available to COBI and will be used solely for this purpose. You can decide not to answer a question at any time during this interview.

Date of the interview

Day: | | | Month | | | Year | | |

A. Name of interviewee: ________________

B. Name of interviewer: _____________

C. Place where the interview took place: ____________________

I. General information

1.1. Age: **15 / 16 / 17 / 18 / 19 / 20 / 21 / 22 / 23 / 24 / 25 / 26 / 27 / 28 / 29**

1.2. Civil status or married status: **Single** 01 **/ Free Union** 02 **/ Married** 03 **/ Other** 04

1.3. Sex: **Male** 01 **/ Female** 02

1.4. What is your place of origin? Location______________________

1.5. How many children do you have? **00 / 01 / 02 / 03 / 04**

1.5.1 At what age did you have your first child? **15 / 16 / 17 / 18 / 19 / 20 / 21 / 22 / 23 / 24**

1.6. Do you belong to an ethic group? **Yes** 01 **/ No** 00

1.6.1 Is Yes, which one?

II. Education, Health, Activities and information access

2.1 What is your education or schooling? **None** 01 **/ Elementary School** 02 **/ Unfinished Elementary School 03 / Junior High School** 04 **/ Unfinished Junior School** 05 **/High School** 06 **/Unfinished High School** 07 **/Bachelor's Degree** 08 **/Unfinished Bachelor´s Degree** 09 **/Maters Degree** 10

2.2 Are you currently going to school? Yes 01 / **No** 00 / **Other** (in admission process)

2.2.1 If No, why?

2.3 In case of illness, where do you usually attend? **IMSS/ISSTE** 01 / **IMSABI** 02 / **Private** 03 / **Pharmacy** 04 / **Traditional Medicine** 05 / **Other** 06

2.4.Apart from fishing, do you have any other economic activities? **Yes** 01 / **No** 00

2.4.1. If Yes, which ones? 2.4.2 When?

2.5 How do you connect to the internet? **Prepaid** 01 / **Occasional recharges** 02 / **Modem** 03 / **Ciber coffe** 04 / **Community** 05

2.6 Mention the two social media you used the most

Facebook 01 / **Instagram** 02 / **YouTube** 03 / **WhatsApp** 04 / **None** 05 / **Other** 06

2.6.1 What do you use them for? 2.6.2 What information do you consult in them?

III. Family

3.1 With whom do you live with? Parents 01 / **Alone** 02 / **Partner** 03 / **Friends** 04 / **Other** 05

3.2 What is your parents' education level? **None** 01 / **Elementary School** 02 / **Unfinished Elementary School 03** / **Junior High School** 04 / **Unfinished Junior School** 05 /**High School** 06 /**Unfinished High School** 07 /**Bachelor's Degree** 08 /**Unfinished Bachelor´s Degree** 09 /**Maters Degree** 10

3.3 Where are your parents from?

3.4 What do your parents do?

IV. Aspirations and Expectations

4.1 Besides fishing, have you tried to get another job? **Yes 01** / **No** 00

4.1.1 If Yes, in what?

4.1.1.1 Did you get it? **Yes** 01 / **No** 00

4.1.2 If **No**, why do you think you did not get it?

If you were completely free to choose the best for you	***Sometimes we can't do what we want the most, thinking about your current reality***
4.2 What would you do after finishing your studies?	4.3 What can you really do after finishing your studies?
4.4 What is the job you would like to have for most of your life?	4.5 What job do you really hope to be able to have for most of your life?

4.6 If you were completely free to choose the best for yourself, where would you live?	4.7 Where do you really hope you can live?
4.8 Would you like to participate in fishing in ...<br>4.8.1. Five years? **Yes** 01 / **No** 00<br>4.8.2. Twenty years? **Yes** 01 / **No** 00	4.9. Do you think you can participate in fishing in…<br>4.9.1. Five years? **Yes** 01 / **No** 00<br>4.9.2. Twenty years? **No** 01 / **No** 00

V. Fishing activity

5.1 What is your job in fishing?

5.1.1 For direct fishermen, what species do you fish and with what fishing gear?

5.2 Tell me about your history and your family in fishing. How, with whom and in what way have you participated in the fishing activity throughout your life?

**Age**	**Fishing on the shores / lagoons**	**Disembarkation**	**Equipment maintenance**	**Administration**	**Process**	**Distribution**	**Sell**	**Waiter / companion**	**Fisherman**	**Captain**	**Cooperative Leader**	**Permittee**	**None**
	01	02	03	04	05	07	06	07	08	09	10	11	12
5.2.1 Childhood <12													
5.2.2 Adolescence12-19													
5.2.3 Youth 20-29													
5.2.4 Father													
5.2.5 Mother													
5.2.6 Other family member													

VI. Motivation - *Why do you do it?*

6.1 What do you like about fishing??

6.2 What is the main benefit that you receive from fishing?

6.3 What do your parents think about you working in fishing?

6.4 Are you a member of a fishing organization? **Yes Si** 01 / **No** 00

6.4.1 If **yes**, is your opinion taken into account in the organization? **Yes** 01 / **No** 00

6.4.2 If **No**, why?

6.4.2.1 Would you like to become part of a fishing organization?? Yes 01 / **No** 00

6.5 Do you consider yourself a fisherman?? **Yes** 01 / **No** 00

6.5.2 If **yes**, what does it mean to you be a young person who worked in fishing?

6.5.3 If **No**, why?

6.6 Do you see yourself as a fishing leader? **Yes 01** / **No** 00

6.6.1 If **yes**, what do you think is your greatest ability to be a leader?

6.7 What do you think would it take to encourage youth participation in fishing?

VII. Sustainability

7.1 What does the sustainable use / conservation of natural resources mean to you?

7.2 Have you received any courses, training or are you currently participating in a marine conservation or sustainable fisheries management project? **Yes** 01 / **No** 00

7.2.1 If **Yes**, On what topic? with who?

7.2.2 With what you have learned, have you implemented an activity or project in your region / community / fishery?

7.2.3 Have you talked about it with someone who has not attended the courses or trainings? Yes 01 / No 00

7.2.3.1 With whom?

7.3 How has fishing changed as your grandparents and father have told you? How do you think it will be in the future?

7.4 What can previous generations of fishermen teach you about conserving fish resources?

7.5 You as a young man or women, what would you teach the generation of older fishermen?

7.6 From the list of eight topics to improve fishing that we send you, can you help us organize them in order of importance? where is the most important and 8 the less important

( ) Value chains and markets

( ) Institutional coordination

( ) Natural disasters and climate change

( ) Social development, employment and decent work

( ) Gender equality

( ) Sustainable resource and management strategies

( ) Responsible governance

( ) Information, communication and science

7.7 Of the three that we consider the most important, you can answer:

A. Value chains and markets

A.1 How important is it for you to promote **exports** and **local trade**? And why?

A.2 Do you know how far your product goes?? ¿who distribute it? If someone else distributes it, why doesn't the fishing community do it?

A.3 Do you feel that what they pay you for your product is fair? If **No**, why are you selling in this way? Where else could they sell it? What would you propose to get a better price?

B. Institutional coordination

B.1 Mention the authorities involved in fishing in your community?

B.2 What do you propose to improve **collaboration** between authorities and fishermen?

B.3 Is there any coordination between fishermen? If yes, how do you organize to go out fishing?

C. Natural disasters and climate change

C.1 What do you think is climate change? How does it affect fishing?

C.2 In your community, are you prepared to face the natural disasters and human impact on the fishing activity? How could your community improve their ability to cope with natural disasters?

D. Social development, employment and decent work

D.1 What do you think is needed to improve safety of fishermen at sea?

D.2 Do you think that the families of the fishermen in your community are guaranteed education, decent employment and sufficient livelihood? why? What could be changed to improve?

D.3 If we had to temporarily suspend fishing, what other activities do you think these families could work?

E. Gender equity

E.1 ¿ What do you think of when I say "gender equality" in fishing? Do you have any examples of inequality between men and women in your fishing?

E.2 Do women and men have the same right to participate in all fishing activities equally?

E.3 In your experience, if men and women did the same activity, would they earn the same? Why?

F. Responsible governance

F.1 Do you think we should share the use of the sea? Who should have rights over the sea?

F.2 Do you identify any conflict between the authorities and fishermen? Do you have any suggestions that can help resolve those conflicts?

G. Sustainable resource and management strategies

G.1In your opinion, what should be the measures / strategies to conserve and sustainably use marine ecosystems and resources? What would you like to change about the way your community fishes? Why?

G.2 What effects do you think the ANPs, the monitoring, the fishing exclusion zones have had on fishing?

G.3 To what extent is the opinion of fishermen taken into account to establish fishing regulations? How would you promote greater participation in fisheries management?

G.4 Regarding closures, surveillance and government support, what should be the government's commitment and what should be the fishermen?

H. Information, communication and science

H.1 In your opinion, why is fishermen knowledge important? What would you like to keep or learn from it?

H.2 What have you learned about fishing and where did you learn it?

H.3 What would you like to learn about fisheries resources and the sea / marine ecosystem? How would you like to learn it?

Appendix S3

Infographic used to help interviewees in determining the level of importance attributed to different processes of SSF sustainability.

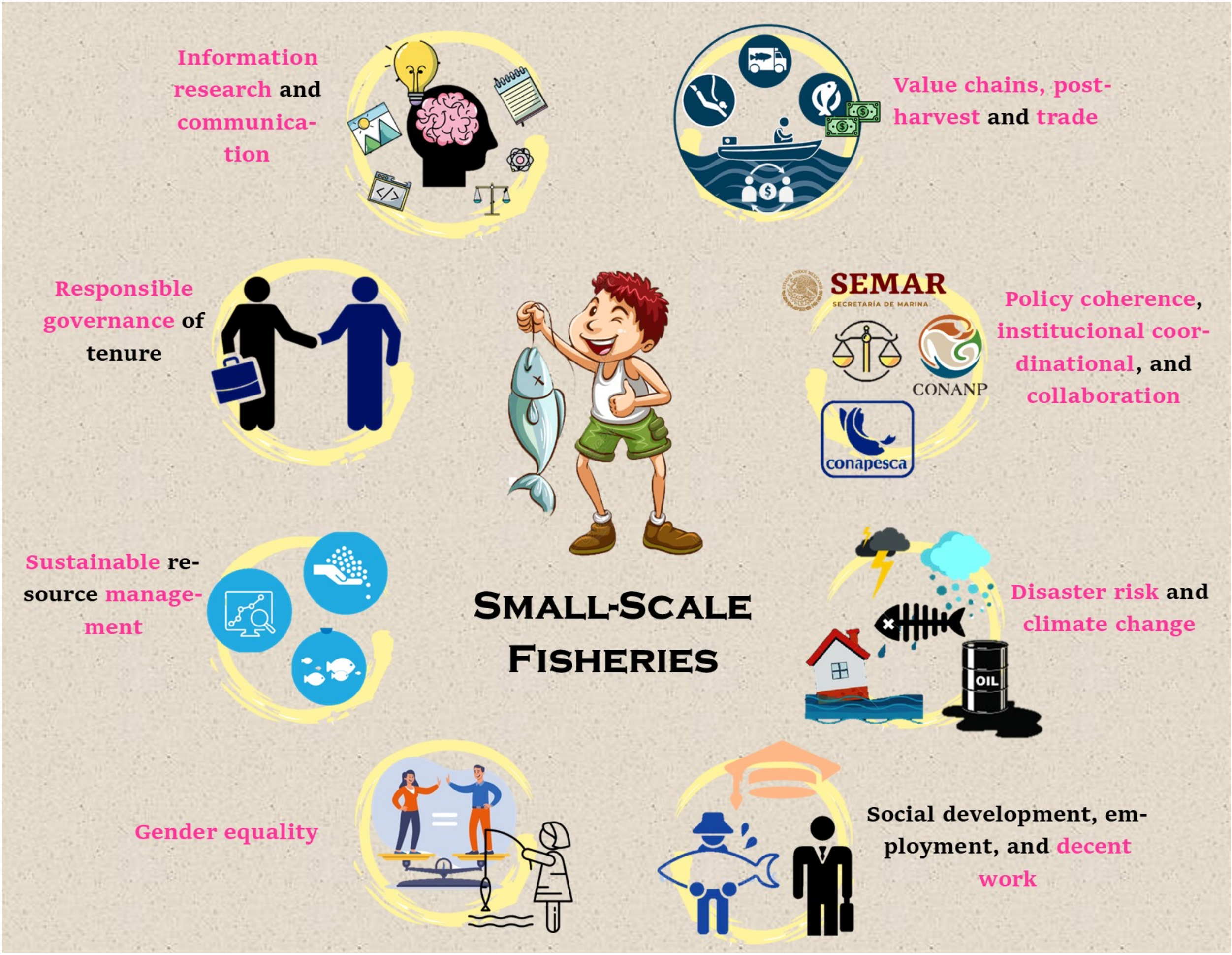

Supplement: Supplementary file 1 — Supplementary file1 (PDF 1928 kb) [file 13280_2021_1639_MOESM1_ESM.pdf]
